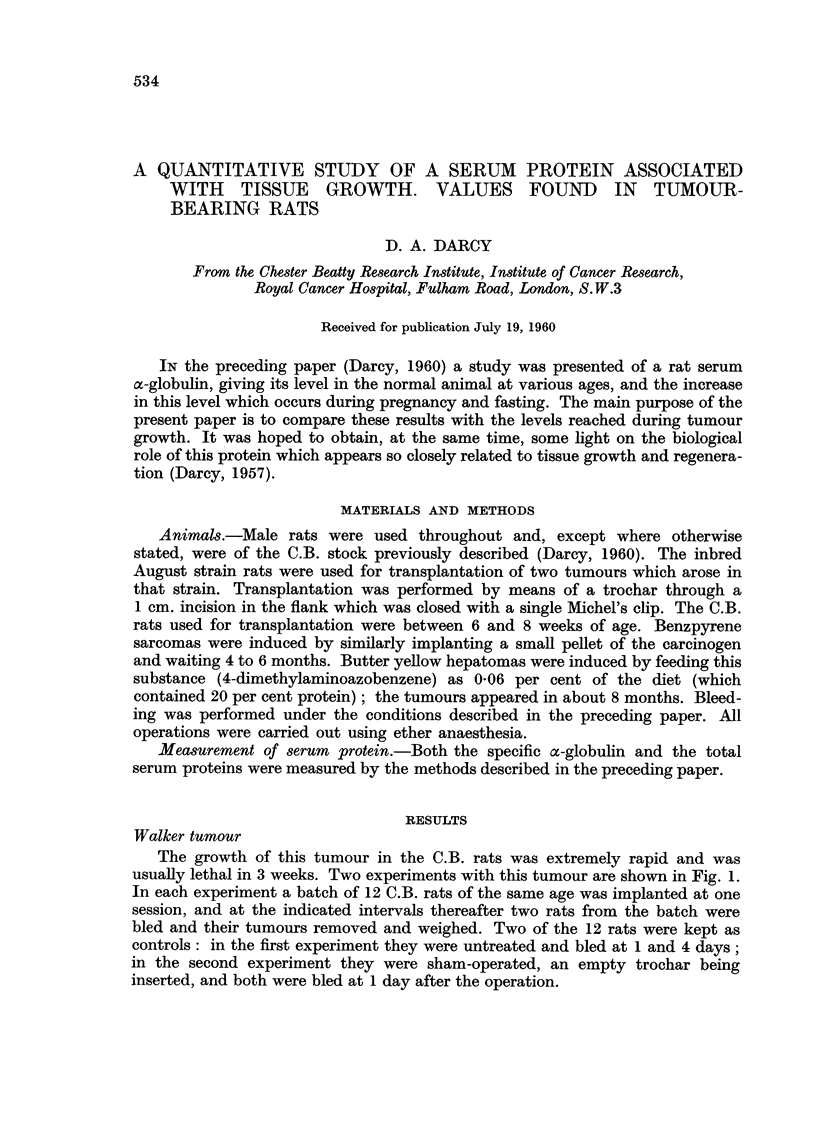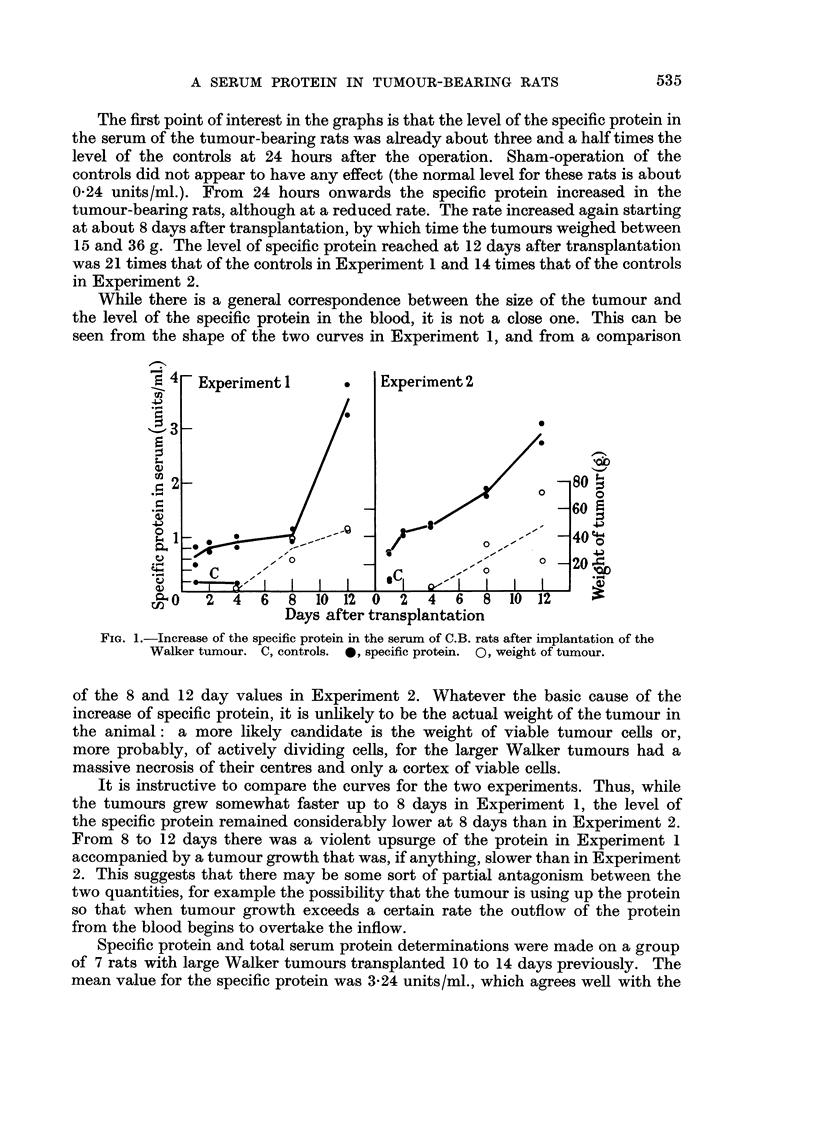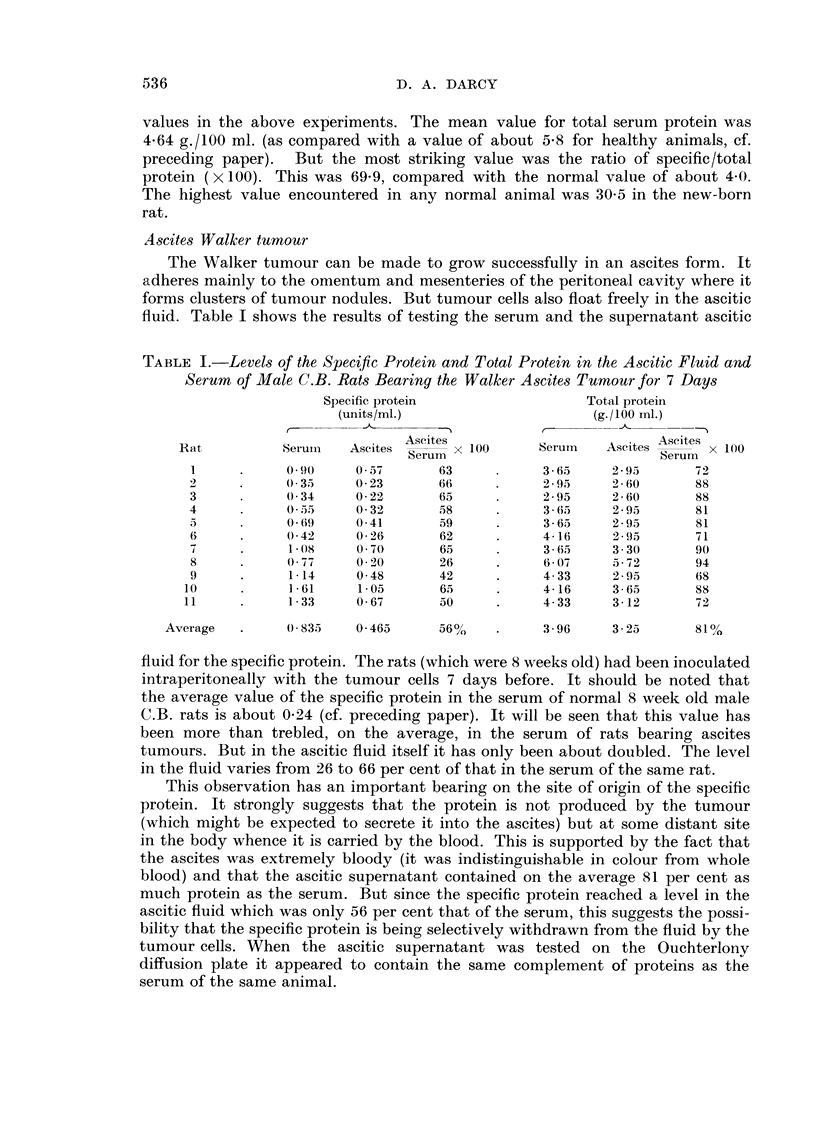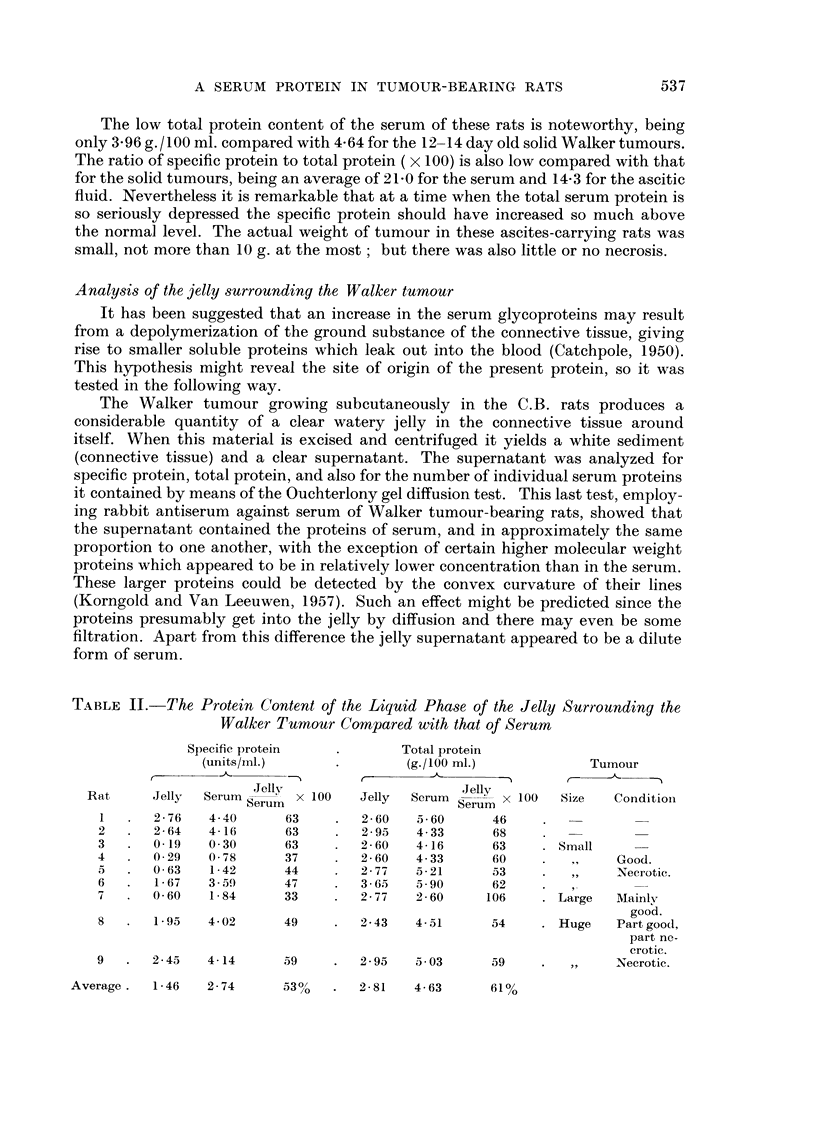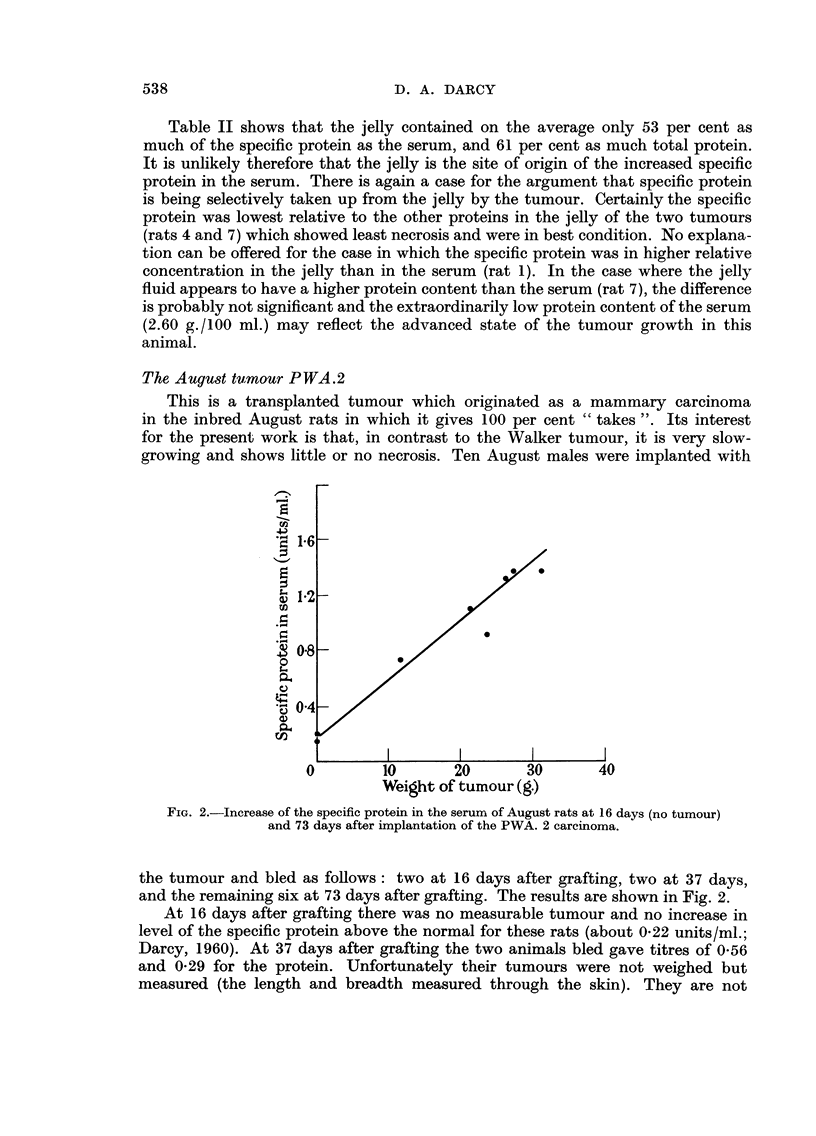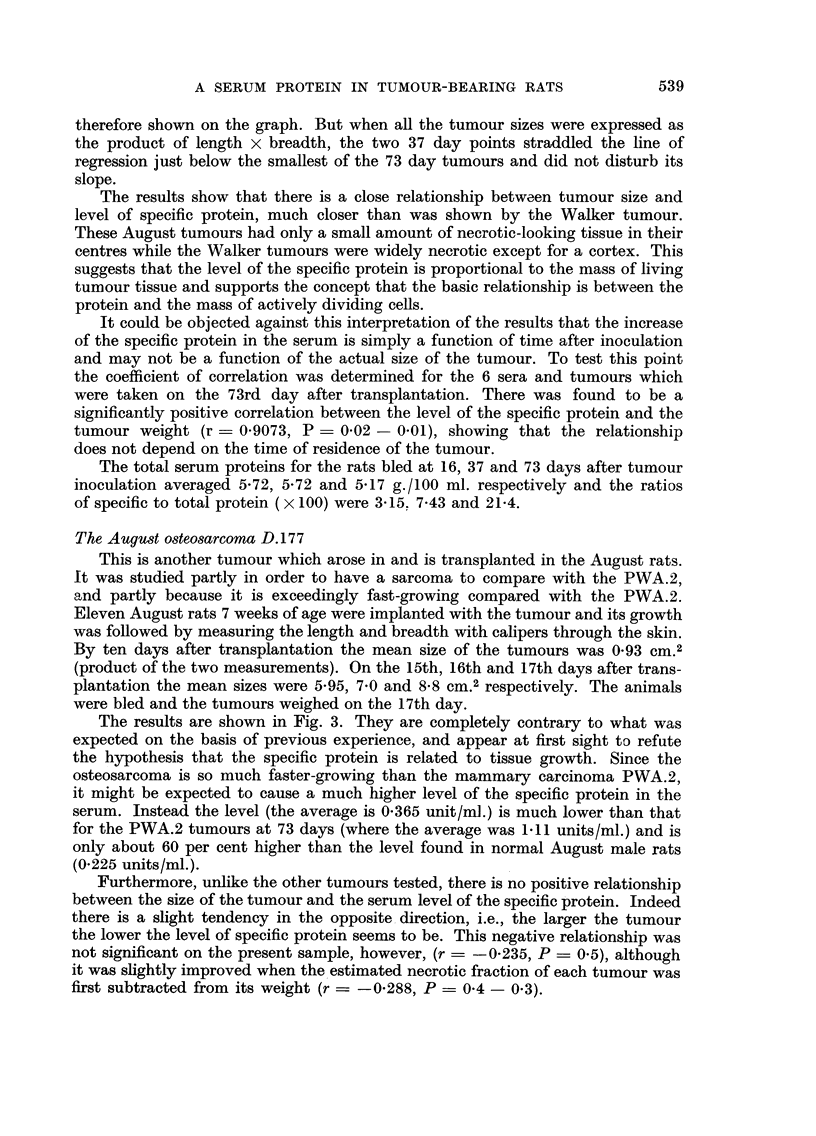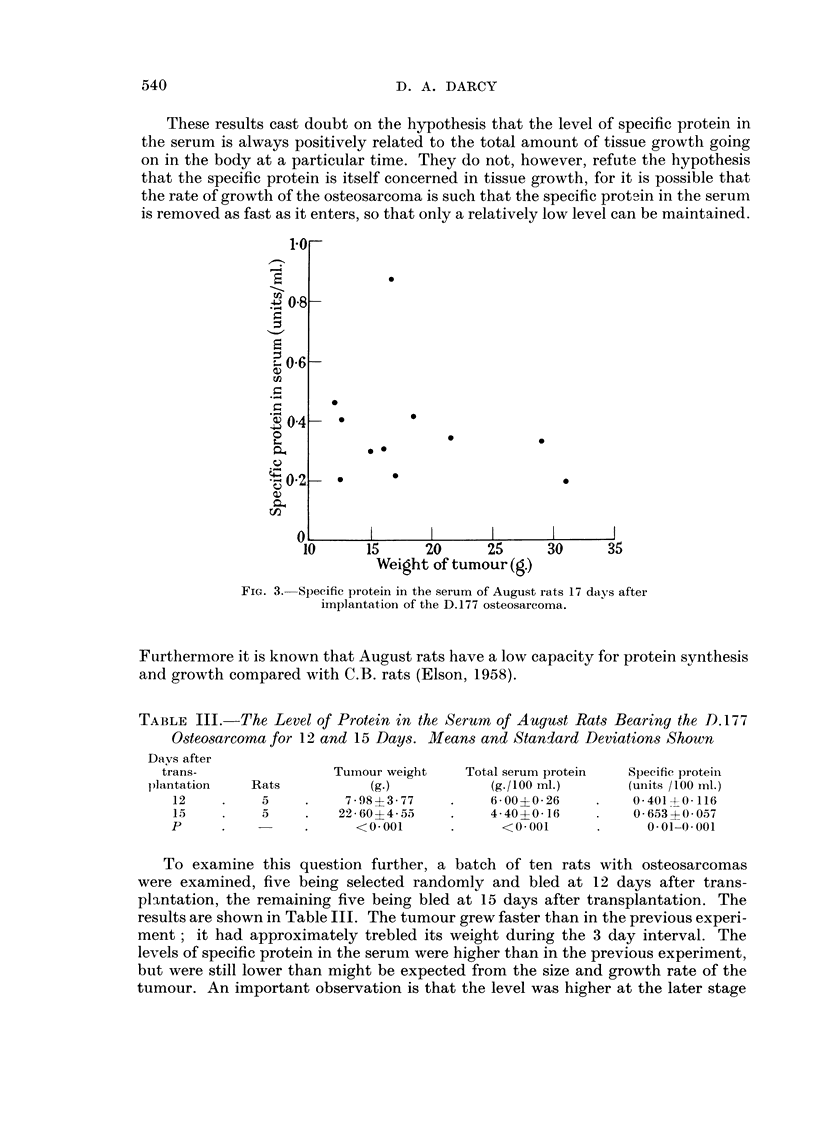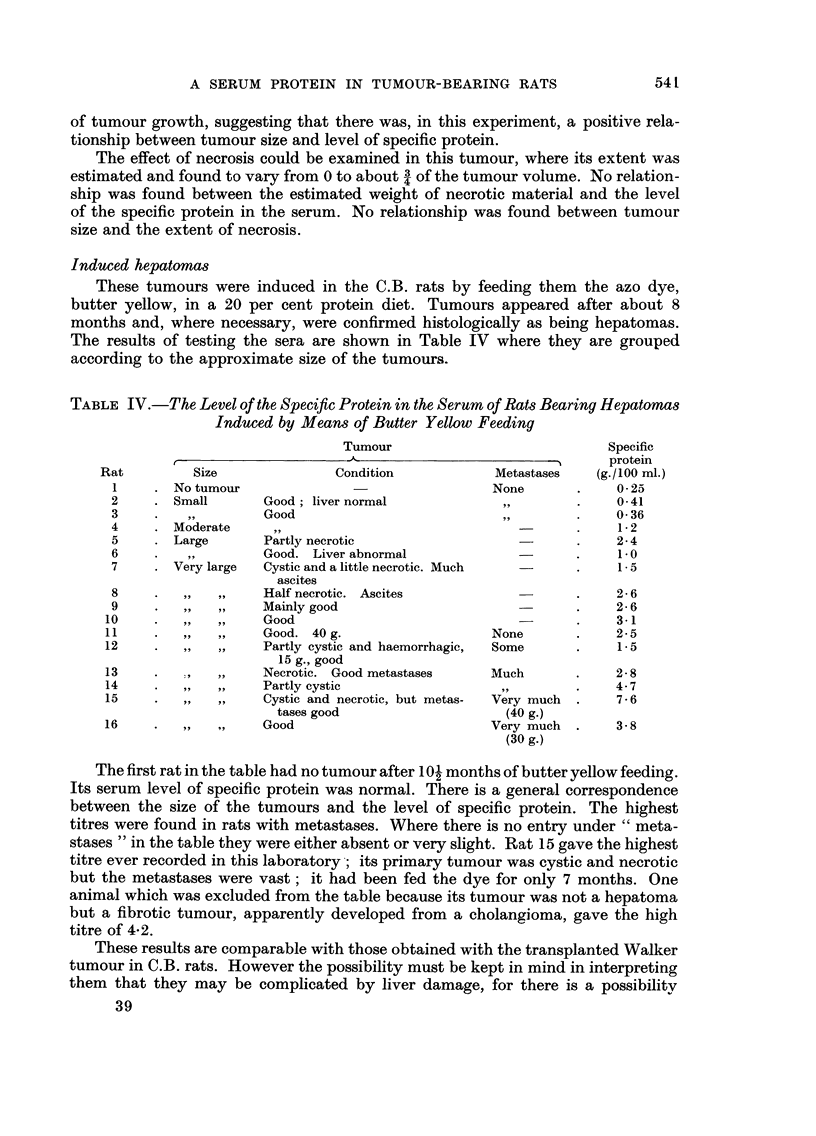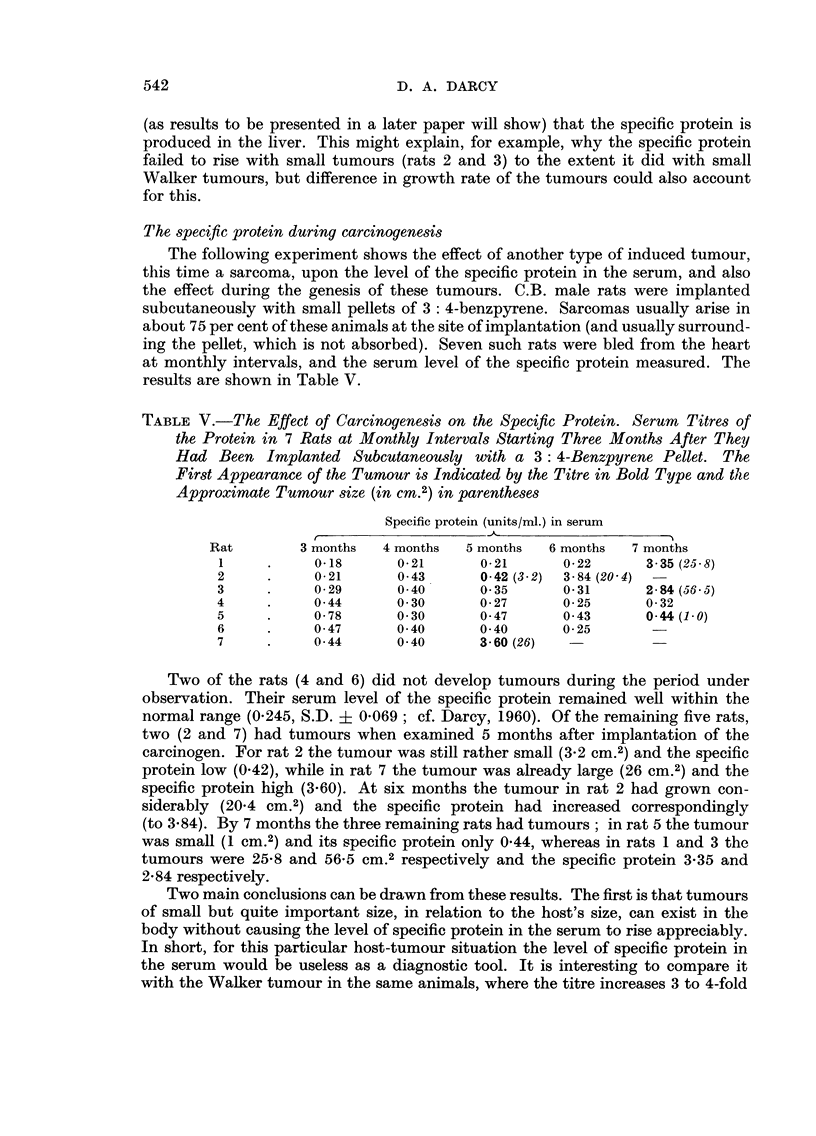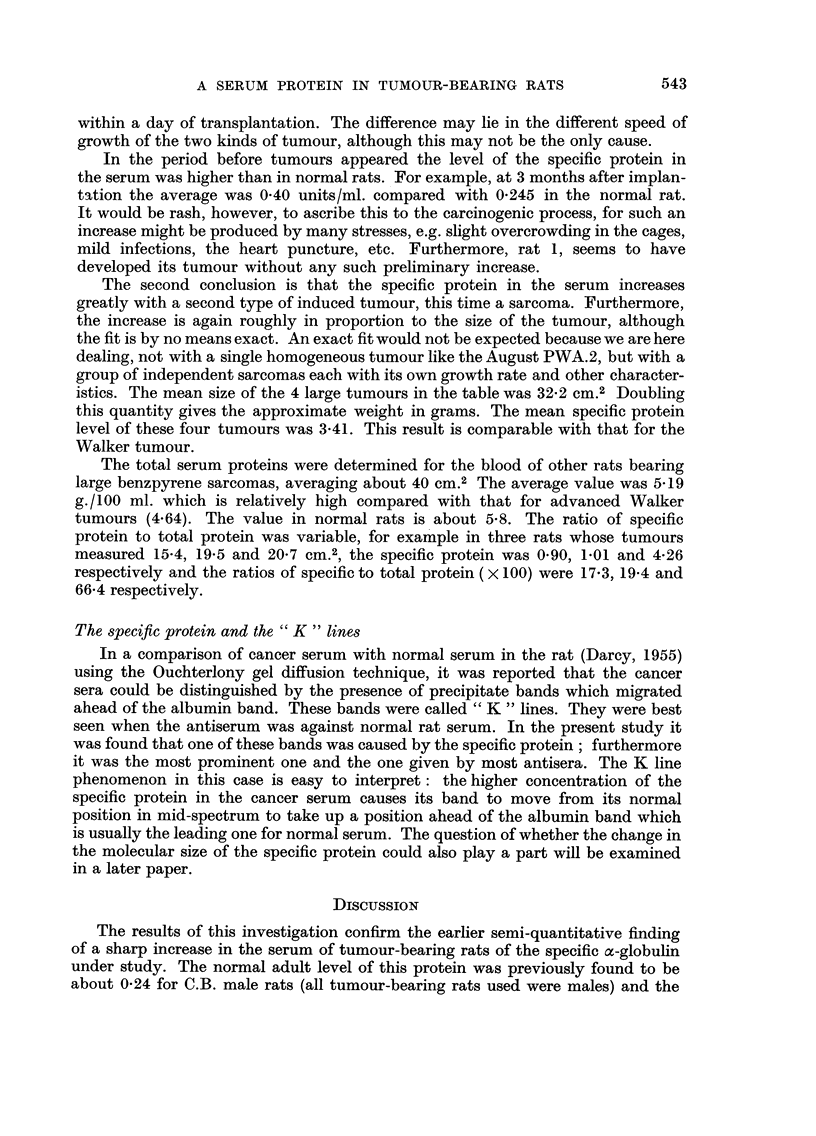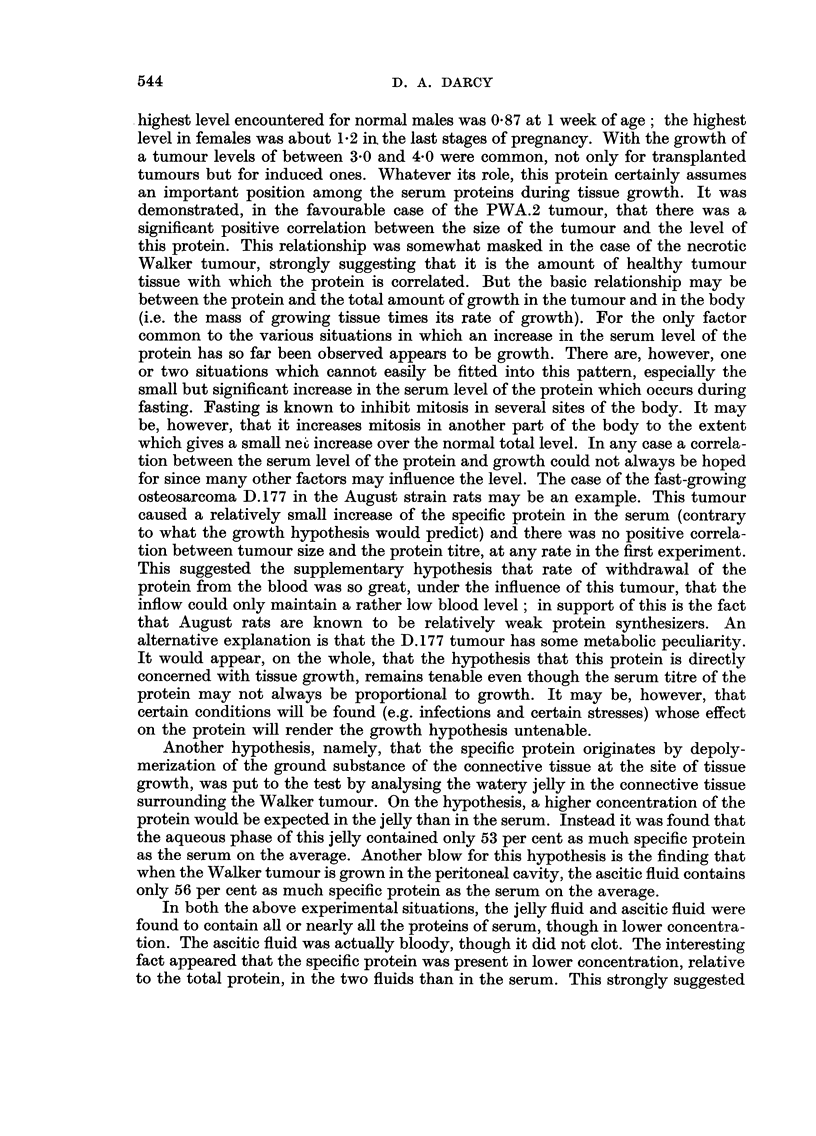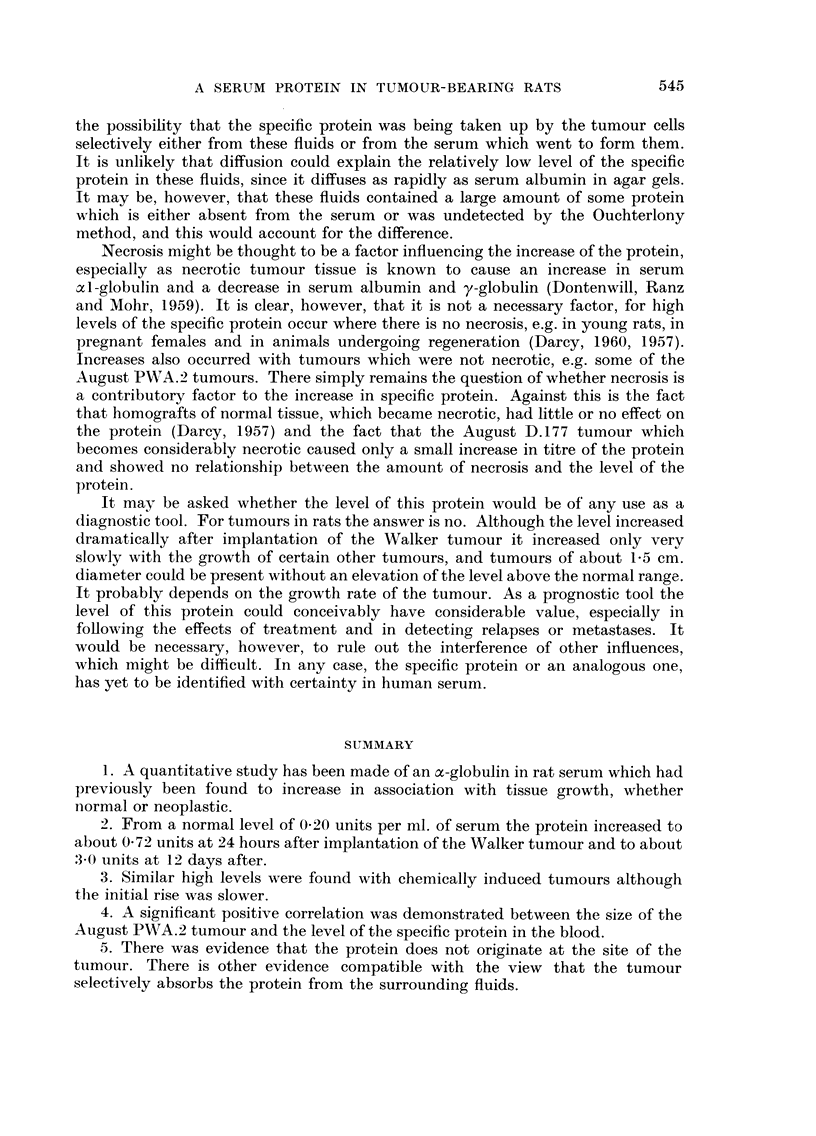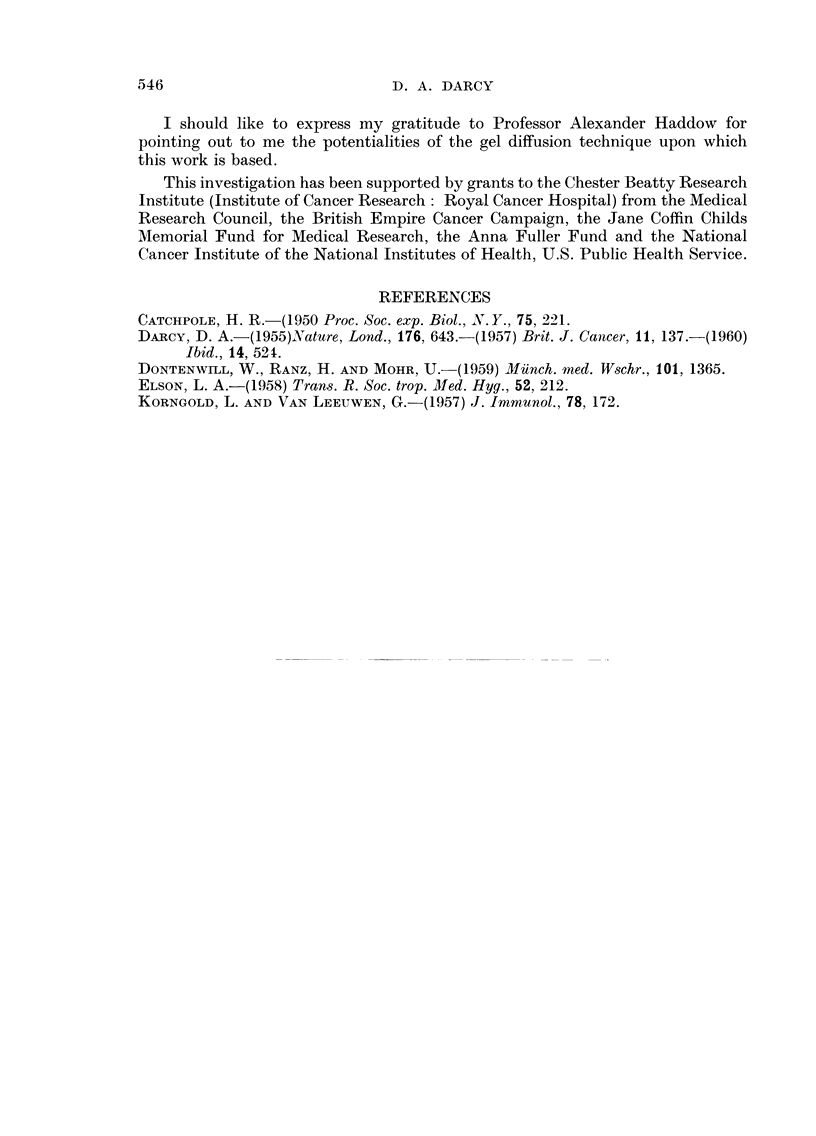# A Quantitative Study of a Serum Protein Associated with Tissue Growth. Values found in Tumour-bearing Rats

**DOI:** 10.1038/bjc.1960.60

**Published:** 1960-09

**Authors:** D. A. Darcy


### A QUANTITATIVE STUDY OF A SERUM PROTEIN ASSOCIATED WITH TISSUE GROWTH. VALUES FOUND IN TUMOUR-BEARING RATS

D. A. DARCY

*From the Chester Beatty Research Institute, Institute of Cancer Research, Royal Cancer Hospital, Fulham Road, London, S.W.3*

Received for publication July 19, 1960

IN the preceding paper (Darcy, 1960) a study was presented of a rat serum $\alpha$-globulin, giving its level in the normal animal at various ages, and the increase in this level which occurs during pregnancy and fasting. The main purpose of the present paper is to compare these results with the levels reached during tumour growth. It was hoped to obtain, at the same time, some light on the biological role of this protein which appears so closely related to tissue growth and regeneration (Darcy, 1957).

### MATERIALS AND METHODS

*Animals.*—Male rats were used throughout and, except where otherwise stated, were of the C.B. stock previously described (Darcy, 1960). The inbred August strain rats were used for transplantation of two tumours which arose in that strain. Transplantation was performed by means of a trochar through a 1 cm. incision in the flank which was closed with a single Michel's clip. The C.B. rats used for transplantation were between 6 and 8 weeks of age. Benzpyrene sarcomas were induced by similarly implanting a small pellet of the carcinogen and waiting 4 to 6 months. Butter yellow hepatomas were induced by feeding this substance (4-dimethylaminoazobenzene) as 0·06 per cent of the diet (which contained 20 per cent protein) ; the tumours appeared in about 8 months. Bleeding was performed under the conditions described in the preceding paper. All operations were carried out using ether anaesthesia.

*Measurement of serum protein.*—Both the specific $\alpha$-globulin and the total serum proteins were measured by the methods described in the preceding paper.

### RESULTS

### *Walker tumour*

The growth of this tumour in the C.B. rats was extremely rapid and was usually lethal in 3 weeks. Two experiments with this tumour are shown in Fig. 1. In each experiment a batch of 12 C.B. rats of the same age was implanted at one session, and at the indicated intervals thereafter two rats from the batch were bled and their tumours removed and weighed. Two of the 12 rats were kept as controls : in the first experiment they were untreated and bled at 1 and 4 days ; in the second experiment they were sham-operated, an empty trochar being inserted, and both were bled at 1 day after the operation.

The first point of interest in the graphs is that the level of the specific protein in the serum of the tumour-bearing rats was already about three and a half times the level of the controls at 24 hours after the operation. Sham-operation of the controls did not appear to have any effect (the normal level for these rats is about 0·24 units/ml.). From 24 hours onwards the specific protein increased in the tumour-bearing rats, although at a reduced rate. The rate increased again starting at about 8 days after transplantation, by which time the tumours weighed between 15 and 36 g. The level of specific protein reached at 12 days after transplantation was 21 times that of the controls in Experiment 1 and 14 times that of the controls in Experiment 2.

While there is a general correspondence between the size of the tumour and the level of the specific protein in the blood, it is not a close one. This can be seen from the shape of the two curves in Experiment 1, and from a comparison

FIG. 1.—Increase of the specific protein in the serum of C.B. rats after implantation of the Walker tumour. C, controls. ●, specific protein. ○, weight of tumour.

of the 8 and 12 day values in Experiment 2. Whatever the basic cause of the increase of specific protein, it is unlikely to be the actual weight of the tumour in the animal: a more likely candidate is the weight of viable tumour cells or, more probably, of actively dividing cells, for the larger Walker tumours had a massive necrosis of their centres and only a cortex of viable cells.

It is instructive to compare the curves for the two experiments. Thus, while the tumours grew somewhat faster up to 8 days in Experiment 1, the level of the specific protein remained considerably lower at 8 days than in Experiment 2. From 8 to 12 days there was a violent upsurge of the protein in Experiment 1 accompanied by a tumour growth that was, if anything, slower than in Experiment 2. This suggests that there may be some sort of partial antagonism between the two quantities, for example the possibility that the tumour is using up the protein so that when tumour growth exceeds a certain rate the outflow of the protein from the blood begins to overtake the inflow.

Specific protein and total serum protein determinations were made on a group of 7 rats with large Walker tumours transplanted 10 to 14 days previously. The mean value for the specific protein was 3·24 units/ml., which agrees well with the values in the above experiments. The mean value for total serum protein was 4·64 g./100 ml. (as compared with a value of about 5·8 for healthy animals, cf. preceding paper). But the most striking value was the ratio of specific/total protein (×100). This was 69·9, compared with the normal value of about 4·0. The highest value encountered in any normal animal was 30·5 in the new-born rat.

*Ascites Walker tumour*

The Walker tumour can be made to grow successfully in an ascites form. It adheres mainly to the omentum and mesenteries of the peritoneal cavity where it forms clusters of tumour nodules. But tumour cells also float freely in the ascitic fluid. Table I shows the results of testing the serum and the supernatant ascitic

TABLE I.—*Levels of the Specific Protein and Total Protein in the Ascitic Fluid and Serum of Male C.B. Rats Bearing the Walker Ascites Tumour for 7 Days*

| Rat | Specific protein (units/ml.) | | | Total protein (g./100 ml.) | | |
|---|---|---|---|---|---|---|
| | Serum | Ascites | $\frac{\text{Ascites}}{\text{Serum}} \times 100$ | Serum | Ascites | $\frac{\text{Ascites}}{\text{Serum}} \times 100$ |
| 1 | 0·90 | 0·57 | 63 | 3·65 | 2·95 | 72 |
| 2 | 0·35 | 0·23 | 66 | 2·95 | 2·60 | 88 |
| 3 | 0·34 | 0·22 | 65 | 2·95 | 2·60 | 88 |
| 4 | 0·55 | 0·32 | 58 | 3·65 | 2·95 | 81 |
| 5 | 0·69 | 0·41 | 59 | 3·65 | 2·95 | 81 |
| 6 | 0·42 | 0·26 | 62 | 4·16 | 2·95 | 71 |
| 7 | 1·08 | 0·70 | 65 | 3·65 | 3·30 | 90 |
| 8 | 0·77 | 0·20 | 26 | 6·07 | 5·72 | 94 |
| 9 | 1·14 | 0·48 | 42 | 4·33 | 2·95 | 68 |
| 10 | 1·61 | 1·05 | 65 | 4·16 | 3·65 | 88 |
| 11 | 1·33 | 0·67 | 50 | 4·33 | 3·12 | 72 |
| Average | 0·835 | 0·465 | 56% | 3·96 | 3·25 | 81% |

fluid for the specific protein. The rats (which were 8 weeks old) had been inoculated intraperitoneally with the tumour cells 7 days before. It should be noted that the average value of the specific protein in the serum of normal 8 week old male C.B. rats is about 0·24 (cf. preceding paper). It will be seen that this value has been more than trebled, on the average, in the serum of rats bearing ascites tumours. But in the ascitic fluid itself it has only been about doubled. The level in the fluid varies from 26 to 66 per cent of that in the serum of the same rat.

This observation has an important bearing on the site of origin of the specific protein. It strongly suggests that the protein is not produced by the tumour (which might be expected to secrete it into the ascites) but at some distant site in the body whence it is carried by the blood. This is supported by the fact that the ascites was extremely bloody (it was indistinguishable in colour from whole blood) and that the ascitic supernatant contained on the average 81 per cent as much protein as the serum. But since the specific protein reached a level in the ascitic fluid which was only 56 per cent that of the serum, this suggests the possibility that the specific protein is being selectively withdrawn from the fluid by the tumour cells. When the ascitic supernatant was tested on the Ouchterlony diffusion plate it appeared to contain the same complement of proteins as the serum of the same animal.

The low total protein content of the serum of these rats is noteworthy, being only 3·96 g./100 ml. compared with 4·64 for the 12–14 day old solid Walker tumours. The ratio of specific protein to total protein ( × 100) is also low compared with that for the solid tumours, being an average of 21·0 for the serum and 14·3 for the ascitic fluid. Nevertheless it is remarkable that at a time when the total serum protein is so seriously depressed the specific protein should have increased so much above the normal level. The actual weight of tumour in these ascites-carrying rats was small, not more than 10 g. at the most ; but there was also little or no necrosis.

*Analysis of the jelly surrounding the Walker tumour*

It has been suggested that an increase in the serum glycoproteins may result from a depolymerization of the ground substance of the connective tissue, giving rise to smaller soluble proteins which leak out into the blood (Catchpole, 1950). This hypothesis might reveal the site of origin of the present protein, so it was tested in the following way.

The Walker tumour growing subcutaneously in the C.B. rats produces a considerable quantity of a clear watery jelly in the connective tissue around itself. When this material is excised and centrifuged it yields a white sediment (connective tissue) and a clear supernatant. The supernatant was analyzed for specific protein, total protein, and also for the number of individual serum proteins it contained by means of the Ouchterlony gel diffusion test. This last test, employing rabbit antiserum against serum of Walker tumour-bearing rats, showed that the supernatant contained the proteins of serum, and in approximately the same proportion to one another, with the exception of certain higher molecular weight proteins which appeared to be in relatively lower concentration than in the serum. These larger proteins could be detected by the convex curvature of their lines (Korngold and Van Leeuwen, 1957). Such an effect might be predicted since the proteins presumably get into the jelly by diffusion and there may even be some filtration. Apart from this difference the jelly supernatant appeared to be a dilute form of serum.

TABLE II.—*The Protein Content of the Liquid Phase of the Jelly Surrounding the Walker Tumour Compared with that of Serum*

| | Specific protein (units/ml.) | | | Total protein (g./100 ml.) | | | Tumour | |
|---|---|---|---|---|---|---|---|---|
| Rat | Jelly | Serum | $\frac{\text{Jelly}}{\text{Serum}} \times 100$ | Jelly | Serum | $\frac{\text{Jelly}}{\text{Serum}} \times 100$ | Size | Condition |
| 1 | 2·76 | 4·40 | 63 | 2·60 | 5·60 | 46 | — | — |
| 2 | 2·64 | 4·16 | 63 | 2·95 | 4·33 | 68 | — | — |
| 3 | 0·19 | 0·30 | 63 | 2·60 | 4·16 | 63 | Small | — |
| 4 | 0·29 | 0·78 | 37 | 2·60 | 4·33 | 60 | ,, | Good. |
| 5 | 0·63 | 1·42 | 44 | 2·77 | 5·21 | 53 | ,, | Necrotic. |
| 6 | 1·67 | 3·59 | 47 | 3·65 | 5·90 | 62 | ,, | — |
| 7 | 0·60 | 1·84 | 33 | 2·77 | 2·60 | 106 | Large | Mainly good. |
| 8 | 1·95 | 4·02 | 49 | 2·43 | 4·51 | 54 | Huge | Part good, part necrotic. |
| 9 | 2·45 | 4·14 | 59 | 2·95 | 5·03 | 59 | ,, | Necrotic. |
| Average | 1·46 | 2·74 | 53% | 2·81 | 4·63 | 61% | | |

Table II shows that the jelly contained on the average only 53 per cent as much of the specific protein as the serum, and 61 per cent as much total protein. It is unlikely therefore that the jelly is the site of origin of the increased specific protein in the serum. There is again a case for the argument that specific protein is being selectively taken up from the jelly by the tumour. Certainly the specific protein was lowest relative to the other proteins in the jelly of the two tumours (rats 4 and 7) which showed least necrosis and were in best condition. No explanation can be offered for the case in which the specific protein was in higher relative concentration in the jelly than in the serum (rat 1). In the case where the jelly fluid appears to have a higher protein content than the serum (rat 7), the difference is probably not significant and the extraordinarily low protein content of the serum (2.60 g./100 ml.) may reflect the advanced state of the tumour growth in this animal.

*The August tumour PWA.2*

This is a transplanted tumour which originated as a mammary carcinoma in the inbred August rats in which it gives 100 per cent " takes ". Its interest for the present work is that, in contrast to the Walker tumour, it is very slow-growing and shows little or no necrosis. Ten August males were implanted with

FIG. 2.—Increase of the specific protein in the serum of August rats at 16 days (no tumour) and 73 days after implantation of the PWA. 2 carcinoma.

the tumour and bled as follows : two at 16 days after grafting, two at 37 days, and the remaining six at 73 days after grafting. The results are shown in Fig. 2.

At 16 days after grafting there was no measurable tumour and no increase in level of the specific protein above the normal for these rats (about 0·22 units/ml.; Darcy, 1960). At 37 days after grafting the two animals bled gave titres of 0·56 and 0·29 for the protein. Unfortunately their tumours were not weighed but measured (the length and breadth measured through the skin). They are not therefore shown on the graph. But when all the tumour sizes were expressed as the product of length × breadth, the two 37 day points straddled the line of regression just below the smallest of the 73 day tumours and did not disturb its slope.

The results show that there is a close relationship between tumour size and level of specific protein, much closer than was shown by the Walker tumour. These August tumours had only a small amount of necrotic-looking tissue in their centres while the Walker tumours were widely necrotic except for a cortex. This suggests that the level of the specific protein is proportional to the mass of living tumour tissue and supports the concept that the basic relationship is between the protein and the mass of actively dividing cells.

It could be objected against this interpretation of the results that the increase of the specific protein in the serum is simply a function of time after inoculation and may not be a function of the actual size of the tumour. To test this point the coefficient of correlation was determined for the 6 sera and tumours which were taken on the 73rd day after transplantation. There was found to be a significantly positive correlation between the level of the specific protein and the tumour weight (r = 0·9073, P = 0·02 — 0·01), showing that the relationship does not depend on the time of residence of the tumour.

The total serum proteins for the rats bled at 16, 37 and 73 days after tumour inoculation averaged 5·72, 5·72 and 5·17 g./100 ml. respectively and the ratios of specific to total protein (×100) were 3·15, 7·43 and 21·4.

*The August osteosarcoma D.*177

This is another tumour which arose in and is transplanted in the August rats. It was studied partly in order to have a sarcoma to compare with the PWA.2, and partly because it is exceedingly fast-growing compared with the PWA.2. Eleven August rats 7 weeks of age were implanted with the tumour and its growth was followed by measuring the length and breadth with calipers through the skin. By ten days after transplantation the mean size of the tumours was 0·93 cm.$^2$ (product of the two measurements). On the 15th, 16th and 17th days after transplantation the mean sizes were 5·95, 7·0 and 8·8 cm.$^2$ respectively. The animals were bled and the tumours weighed on the 17th day.

The results are shown in Fig. 3. They are completely contrary to what was expected on the basis of previous experience, and appear at first sight to refute the hypothesis that the specific protein is related to tissue growth. Since the osteosarcoma is so much faster-growing than the mammary carcinoma PWA.2, it might be expected to cause a much higher level of the specific protein in the serum. Instead the level (the average is 0·365 unit/ml.) is much lower than that for the PWA.2 tumours at 73 days (where the average was 1·11 units/ml.) and is only about 60 per cent higher than the level found in normal August male rats (0·225 units/ml.).

Furthermore, unlike the other tumours tested, there is no positive relationship between the size of the tumour and the serum level of the specific protein. Indeed there is a slight tendency in the opposite direction, i.e., the larger the tumour the lower the level of specific protein seems to be. This negative relationship was not significant on the present sample, however, ($r = -0{\cdot}235$, $P = 0{\cdot}5$), although it was slightly improved when the estimated necrotic fraction of each tumour was first subtracted from its weight ($r = -0{\cdot}288$, $P = 0{\cdot}4 - 0{\cdot}3$).

These results cast doubt on the hypothesis that the level of specific protein in the serum is always positively related to the total amount of tissue growth going on in the body at a particular time. They do not, however, refute the hypothesis that the specific protein is itself concerned in tissue growth, for it is possible that the rate of growth of the osteosarcoma is such that the specific protein in the serum is removed as fast as it enters, so that only a relatively low level can be maintained.

FIG. 3.—Specific protein in the serum of August rats 17 days after implantation of the D.177 osteosarcoma.

Furthermore it is known that August rats have a low capacity for protein synthesis and growth compared with C.B. rats (Elson, 1958).

TABLE III.—*The Level of Protein in the Serum of August Rats Bearing the D.*177 *Osteosarcoma for* 12 *and* 15 *Days. Means and Standard Deviations Shown*

| Days after trans-plantation | Rats | Tumour weight (g.) | Total serum protein (g./100 ml.) | Specific protein (units /100 ml.) |
|---|---|---|---|---|
| 12 | 5 | 7·98±3·77 | 6·00±0·26 | 0·401±0·116 |
| 15 | 5 | 22·60±4·55 | 4·40±0·16 | 0·653±0·057 |
| *P* | — | <0·001 | <0·001 | 0·01–0·001 |

To examine this question further, a batch of ten rats with osteosarcomas were examined, five being selected randomly and bled at 12 days after transplantation, the remaining five being bled at 15 days after transplantation. The results are shown in Table III. The tumour grew faster than in the previous experiment; it had approximately trebled its weight during the 3 day interval. The levels of specific protein in the serum were higher than in the previous experiment, but were still lower than might be expected from the size and growth rate of the tumour. An important observation is that the level was higher at the later stage of tumour growth, suggesting that there was, in this experiment, a positive relationship between tumour size and level of specific protein.

The effect of necrosis could be examined in this tumour, where its extent was estimated and found to vary from 0 to about $\frac{3}{4}$ of the tumour volume. No relationship was found between the estimated weight of necrotic material and the level of the specific protein in the serum. No relationship was found between tumour size and the extent of necrosis.

*Induced hepatomas*

These tumours were induced in the C.B. rats by feeding them the azo dye, butter yellow, in a 20 per cent protein diet. Tumours appeared after about 8 months and, where necessary, were confirmed histologically as being hepatomas. The results of testing the sera are shown in Table IV where they are grouped according to the approximate size of the tumours.

TABLE IV.—*The Level of the Specific Protein in the Serum of Rats Bearing Hepatomas Induced by Means of Butter Yellow Feeding*

| Rat | Tumour: Size | Tumour: Condition | Tumour: Metastases | Specific protein (g./100 ml.) |
|---|---|---|---|---|
| 1 | No tumour | — | None | 0·25 |
| 2 | Small | Good ; liver normal | ,, | 0·41 |
| 3 | ,, | Good | ,, | 0·36 |
| 4 | Moderate | ,, | — | 1·2 |
| 5 | Large | Partly necrotic | — | 2·4 |
| 6 | ,, | Good. Liver abnormal | — | 1·0 |
| 7 | Very large | Cystic and a little necrotic. Much ascites | — | 1·5 |
| 8 | ,, ,, | Half necrotic. Ascites | — | 2·6 |
| 9 | ,, ,, | Mainly good | — | 2·6 |
| 10 | ,, ,, | Good | — | 3·1 |
| 11 | ,, ,, | Good. 40 g. | None | 2·5 |
| 12 | ,, ,, | Partly cystic and haemorrhagic, 15 g., good | Some | 1·5 |
| 13 | ,, ,, | Necrotic. Good metastases | Much | 2·8 |
| 14 | ,, ,, | Partly cystic | ,, | 4·7 |
| 15 | ,, ,, | Cystic and necrotic, but metastases good | Very much (40 g.) | 7·6 |
| 16 | ,, ,, | Good | Very much (30 g.) | 3·8 |

The first rat in the table had no tumour after 10½ months of butter yellow feeding. Its serum level of specific protein was normal. There is a general correspondence between the size of the tumours and the level of specific protein. The highest titres were found in rats with metastases. Where there is no entry under " metastases " in the table they were either absent or very slight. Rat 15 gave the highest titre ever recorded in this laboratory ; its primary tumour was cystic and necrotic but the metastases were vast ; it had been fed the dye for only 7 months. One animal which was excluded from the table because its tumour was not a hepatoma but a fibrotic tumour, apparently developed from a cholangioma, gave the high titre of 4·2.

These results are comparable with those obtained with the transplanted Walker tumour in C.B. rats. However the possibility must be kept in mind in interpreting them that they may be complicated by liver damage, for there is a possibility

(as results to be presented in a later paper will show) that the specific protein is produced in the liver. This might explain, for example, why the specific protein failed to rise with small tumours (rats 2 and 3) to the extent it did with small Walker tumours, but difference in growth rate of the tumours could also account for this.

*The specific protein during carcinogenesis*

The following experiment shows the effect of another type of induced tumour, this time a sarcoma, upon the level of the specific protein in the serum, and also the effect during the genesis of these tumours. C.B. male rats were implanted subcutaneously with small pellets of 3 : 4-benzpyrene. Sarcomas usually arise in about 75 per cent of these animals at the site of implantation (and usually surrounding the pellet, which is not absorbed). Seven such rats were bled from the heart at monthly intervals, and the serum level of the specific protein measured. The results are shown in Table V.

TABLE V.—*The Effect of Carcinogenesis on the Specific Protein. Serum Titres of the Protein in 7 Rats at Monthly Intervals Starting Three Months After They Had Been Implanted Subcutaneously with a 3 : 4-Benzpyrene Pellet. The First Appearance of the Tumour is Indicated by the Titre in Bold Type and the Approximate Tumour size (in cm.$^2$) in parentheses*

| Rat | Specific protein (units/ml.) in serum | | | | |
|---|---|---|---|---|---|
| | 3 months | 4 months | 5 months | 6 months | 7 months |
| 1 | 0·18 | 0·21 | 0·21 | 0·22 | **3·35** (*25·8*) |
| 2 | 0·21 | 0·43 | **0·42** (*3·2*) | 3·84 (*20·4*) | — |
| 3 | 0·29 | 0·40 | 0·35 | 0·31 | **2·84** (*56·5*) |
| 4 | 0·44 | 0·30 | 0·27 | 0·25 | 0·32 |
| 5 | 0·78 | 0·30 | 0·47 | 0·43 | **0·44** (*1·0*) |
| 6 | 0·47 | 0·40 | 0·40 | 0·25 | — |
| 7 | 0·44 | 0·40 | **3·60** (*26*) | — | — |

Two of the rats (4 and 6) did not develop tumours during the period under observation. Their serum level of the specific protein remained well within the normal range (0·245, S.D. ± 0·069 ; cf. Darcy, 1960). Of the remaining five rats, two (2 and 7) had tumours when examined 5 months after implantation of the carcinogen. For rat 2 the tumour was still rather small (3·2 cm.$^2$) and the specific protein low (0·42), while in rat 7 the tumour was already large (26 cm.$^2$) and the specific protein high (3·60). At six months the tumour in rat 2 had grown considerably (20·4 cm.$^2$) and the specific protein had increased correspondingly (to 3·84). By 7 months the three remaining rats had tumours ; in rat 5 the tumour was small (1 cm.$^2$) and its specific protein only 0·44, whereas in rats 1 and 3 the tumours were 25·8 and 56·5 cm.$^2$ respectively and the specific protein 3·35 and 2·84 respectively.

Two main conclusions can be drawn from these results. The first is that tumours of small but quite important size, in relation to the host's size, can exist in the body without causing the level of specific protein in the serum to rise appreciably. In short, for this particular host-tumour situation the level of specific protein in the serum would be useless as a diagnostic tool. It is interesting to compare it with the Walker tumour in the same animals, where the titre increases 3 to 4-fold within a day of transplantation. The difference may lie in the different speed of growth of the two kinds of tumour, although this may not be the only cause.

In the period before tumours appeared the level of the specific protein in the serum was higher than in normal rats. For example, at 3 months after implantation the average was 0·40 units/ml. compared with 0·245 in the normal rat. It would be rash, however, to ascribe this to the carcinogenic process, for such an increase might be produced by many stresses, e.g. slight overcrowding in the cages, mild infections, the heart puncture, etc. Furthermore, rat 1, seems to have developed its tumour without any such preliminary increase.

The second conclusion is that the specific protein in the serum increases greatly with a second type of induced tumour, this time a sarcoma. Furthermore, the increase is again roughly in proportion to the size of the tumour, although the fit is by no means exact. An exact fit would not be expected because we are here dealing, not with a single homogeneous tumour like the August PWA.2, but with a group of independent sarcomas each with its own growth rate and other characteristics. The mean size of the 4 large tumours in the table was 32·2 $cm.^2$ Doubling this quantity gives the approximate weight in grams. The mean specific protein level of these four tumours was 3·41. This result is comparable with that for the Walker tumour.

The total serum proteins were determined for the blood of other rats bearing large benzpyrene sarcomas, averaging about 40 $cm.^2$ The average value was 5·19 g./100 ml. which is relatively high compared with that for advanced Walker tumours (4·64). The value in normal rats is about 5·8. The ratio of specific protein to total protein was variable, for example in three rats whose tumours measured 15·4, 19·5 and 20·7 $cm.^2$, the specific protein was 0·90, 1·01 and 4·26 respectively and the ratios of specific to total protein ($\times$100) were 17·3, 19·4 and 66·4 respectively.

*The specific protein and the " K " lines*

In a comparison of cancer serum with normal serum in the rat (Darcy, 1955) using the Ouchterlony gel diffusion technique, it was reported that the cancer sera could be distinguished by the presence of precipitate bands which migrated ahead of the albumin band. These bands were called " K " lines. They were best seen when the antiserum was against normal rat serum. In the present study it was found that one of these bands was caused by the specific protein ; furthermore it was the most prominent one and the one given by most antisera. The K line phenomenon in this case is easy to interpret : the higher concentration of the specific protein in the cancer serum causes its band to move from its normal position in mid-spectrum to take up a position ahead of the albumin band which is usually the leading one for normal serum. The question of whether the change in the molecular size of the specific protein could also play a part will be examined in a later paper.

### Discussion

The results of this investigation confirm the earlier semi-quantitative finding of a sharp increase in the serum of tumour-bearing rats of the specific $\alpha$-globulin under study. The normal adult level of this protein was previously found to be about 0·24 for C.B. male rats (all tumour-bearing rats used were males) and the highest level encountered for normal males was 0·87 at 1 week of age ; the highest level in females was about 1·2 in the last stages of pregnancy. With the growth of a tumour levels of between 3·0 and 4·0 were common, not only for transplanted tumours but for induced ones. Whatever its role, this protein certainly assumes an important position among the serum proteins during tissue growth. It was demonstrated, in the favourable case of the PWA.2 tumour, that there was a significant positive correlation between the size of the tumour and the level of this protein. This relationship was somewhat masked in the case of the necrotic Walker tumour, strongly suggesting that it is the amount of healthy tumour tissue with which the protein is correlated. But the basic relationship may be between the protein and the total amount of growth in the tumour and in the body (i.e. the mass of growing tissue times its rate of growth). For the only factor common to the various situations in which an increase in the serum level of the protein has so far been observed appears to be growth. There are, however, one or two situations which cannot easily be fitted into this pattern, especially the small but significant increase in the serum level of the protein which occurs during fasting. Fasting is known to inhibit mitosis in several sites of the body. It may be, however, that it increases mitosis in another part of the body to the extent which gives a small net increase over the normal total level. In any case a correlation between the serum level of the protein and growth could not always be hoped for since many other factors may influence the level. The case of the fast-growing osteosarcoma D.177 in the August strain rats may be an example. This tumour caused a relatively small increase of the specific protein in the serum (contrary to what the growth hypothesis would predict) and there was no positive correlation between tumour size and the protein titre, at any rate in the first experiment. This suggested the supplementary hypothesis that rate of withdrawal of the protein from the blood was so great, under the influence of this tumour, that the inflow could only maintain a rather low blood level ; in support of this is the fact that August rats are known to be relatively weak protein synthesizers. An alternative explanation is that the D.177 tumour has some metabolic peculiarity. It would appear, on the whole, that the hypothesis that this protein is directly concerned with tissue growth, remains tenable even though the serum titre of the protein may not always be proportional to growth. It may be, however, that certain conditions will be found (e.g. infections and certain stresses) whose effect on the protein will render the growth hypothesis untenable.

Another hypothesis, namely, that the specific protein originates by depolymerization of the ground substance of the connective tissue at the site of tissue growth, was put to the test by analysing the watery jelly in the connective tissue surrounding the Walker tumour. On the hypothesis, a higher concentration of the protein would be expected in the jelly than in the serum. Instead it was found that the aqueous phase of this jelly contained only 53 per cent as much specific protein as the serum on the average. Another blow for this hypothesis is the finding that when the Walker tumour is grown in the peritoneal cavity, the ascitic fluid contains only 56 per cent as much specific protein as the serum on the average.

In both the above experimental situations, the jelly fluid and ascitic fluid were found to contain all or nearly all the proteins of serum, though in lower concentration. The ascitic fluid was actually bloody, though it did not clot. The interesting fact appeared that the specific protein was present in lower concentration, relative to the total protein, in the two fluids than in the serum. This strongly suggested the possibility that the specific protein was being taken up by the tumour cells selectively either from these fluids or from the serum which went to form them. It is unlikely that diffusion could explain the relatively low level of the specific protein in these fluids, since it diffuses as rapidly as serum albumin in agar gels. It may be, however, that these fluids contained a large amount of some protein which is either absent from the serum or was undetected by the Ouchterlony method, and this would account for the difference.

Necrosis might be thought to be a factor influencing the increase of the protein, especially as necrotic tumour tissue is known to cause an increase in serum $\alpha 1$-globulin and a decrease in serum albumin and $\gamma$-globulin (Dontenwill, Ranz and Mohr, 1959). It is clear, however, that it is not a necessary factor, for high levels of the specific protein occur where there is no necrosis, e.g. in young rats, in pregnant females and in animals undergoing regeneration (Darcy, 1960, 1957). Increases also occurred with tumours which were not necrotic, e.g. some of the August PWA.2 tumours. There simply remains the question of whether necrosis is a contributory factor to the increase in specific protein. Against this is the fact that homografts of normal tissue, which became necrotic, had little or no effect on the protein (Darcy, 1957) and the fact that the August D.177 tumour which becomes considerably necrotic caused only a small increase in titre of the protein and showed no relationship between the amount of necrosis and the level of the protein.

It may be asked whether the level of this protein would be of any use as a diagnostic tool. For tumours in rats the answer is no. Although the level increased dramatically after implantation of the Walker tumour it increased only very slowly with the growth of certain other tumours, and tumours of about 1·5 cm. diameter could be present without an elevation of the level above the normal range. It probably depends on the growth rate of the tumour. As a prognostic tool the level of this protein could conceivably have considerable value, especially in following the effects of treatment and in detecting relapses or metastases. It would be necessary, however, to rule out the interference of other influences, which might be difficult. In any case, the specific protein or an analogous one, has yet to be identified with certainty in human serum.

### SUMMARY

1. A quantitative study has been made of an $\alpha$-globulin in rat serum which had previously been found to increase in association with tissue growth, whether normal or neoplastic.

2. From a normal level of 0·20 units per ml. of serum the protein increased to about 0·72 units at 24 hours after implantation of the Walker tumour and to about 3·0 units at 12 days after.

3. Similar high levels were found with chemically induced tumours although the initial rise was slower.

4. A significant positive correlation was demonstrated between the size of the August PWA.2 tumour and the level of the specific protein in the blood.

5. There was evidence that the protein does not originate at the site of the tumour. There is other evidence compatible with the view that the tumour selectively absorbs the protein from the surrounding fluids.

I should like to express my gratitude to Professor Alexander Haddow for pointing out to me the potentialities of the gel diffusion technique upon which this work is based.

This investigation has been supported by grants to the Chester Beatty Research Institute (Institute of Cancer Research : Royal Cancer Hospital) from the Medical Research Council, the British Empire Cancer Campaign, the Jane Coffin Childs Memorial Fund for Medical Research, the Anna Fuller Fund and the National Cancer Institute of the National Institutes of Health, U.S. Public Health Service.

### REFERENCES

CATCHPOLE, H. R.—(1950 *Proc. Soc. exp. Biol., N.Y.*, **75**, 221.

DARCY, D. A.—(1955)*Nature, Lond.*, **176**, 643.—(1957) *Brit. J. Cancer*, **11**, 137.—(1960) *Ibid.*, **14**, 524.

DONTENWILL, W., RANZ, H. AND MOHR, U.—(1959) *Münch. med. Wschr.*, **101**, 1365.

ELSON, L. A.—(1958) *Trans. R. Soc. trop. Med. Hyg.*, **52**, 212.

KORNGOLD, L. AND VAN LEEUWEN, G.—(1957) *J. Immunol.*, **78**, 172.